# Association of Pathogenic Th17 Cells with the Disease Severity and Its Potential Implication for Biological Treatment Selection in Psoriasis Patients

**DOI:** 10.1155/2020/8065147

**Published:** 2020-07-31

**Authors:** Cristina Aguilar-Flores, Octavio Castro-Escamilla, Elizabeth M. Ortega-Rocha, César Maldonado-García, Fermín Jurado-Santa Cruz, Gibrán Pérez-Montesinos, Alicia Lemini-López, Laura C. Bonifaz

**Affiliations:** ^1^Unidad de Investigación Médica en Inmunoquímica, Hospital de Especialidades, Centro Médico Nacional Siglo XXI (CMNSXXI), Instituto Mexicano del Seguro Social, Mexico City 06720, Mexico; ^2^Departamento de Biología, Facultad de Química, Universidad Nacional Autónoma de México (UNAM), Mexico City 04510, Mexico; ^3^Escuela Nacional de Ciencias Biológicas, Instituto Politécnico Nacional (IPN), Mexico City 11340, Mexico; ^4^Centro Dermatológico “Dr. Ladislao de la Pascua”, Secretaria de Salud de la Ciudad de México, Mexico City 06780, Mexico; ^5^Servicio de Dermatología, Hospital de Especialidades, Centro Médico Nacional Siglo XXI, Instituto Mexicano del Seguro Social, Mexico City 06720, Mexico

## Abstract

Psoriasis is an inflammatory autoimmune disease characterized by cutaneous lesions in plaques. It has been proposed that the immune response has a key role in the disease progression. Particularly, the Th17 cells through IL-17 can contribute to maintain the inflammatory process. The pathogenic Th17 phenotype has been described in human diseases and associated with high severity in inflammatory experimental models. However, it is not clear if the pathogenic phenotype could be present in the skin and peripheral blood as well as its possible association to severity in psoriasis. In the lesional skin, we found high infiltration of Th17 cells and the pathogenic phenotype, finding a correlation between the frequency of Th17 cells and the Psoriasis Area and Severity Index (PASI) score. In peripheral blood, we observed a pool of Th17 lymphocytes with potential to acquire pathogenic features. Interestingly, the percentage of pathogenic Th17 cells (CD4^+^ ROR*γ*t^+^ IFN-*γ*^+^) correlates with disease severity. Moreover, we distinguished three groups of patients based on their IL-17/IFN-*γ* production by Th17 lymphocytes, which seems to be related with a dynamic or stable potential to express these cytokines. Remarkably, we evaluated the cytokine production by Th17 cells as an immunological marker for the adequate selection of biologic therapy. We found that patients analyzed by this immunological approach and treated with antibodies against IL-17 and TNF*α* showed great improvement depicted by reduction in PASI and Dermatology Life Quality Index (DLQI) score as well as the percentage of Body Surface Area (BSA). Altogether, our results highlight the importance of the assessment of the pathogenic phenotype in Th17 cells as an immune personalized analysis with the potential to support the therapy choice in the clinical practice.

## 1. Introduction

Psoriasis is an inflammatory autoimmune skin disease with aberrant keratinocyte differentiation and epidermal thicknesses [1, 2]. The main features are the presence of well-delimitated squamous skin lesions, erythema, and high immune cell infiltration [3, 4]. Without gender preference, the worldwide psoriasis prevalence is estimated between 2 and 3% although some differences are reported according to the study population. It has been observed that prevalence in Taiwan and China is 0.23% and 0.12%, respectively, whereas increased prevalence is reported in Italy (2.1%) and Norway (8.7%) [5, 6].

Psoriasis etiology is not completely understood; however, evidence has shown that the immune response is a key factor in the physiopathology due to the high quantities of inflammatory cytokines such as IL-17 and IL-22 and immune cell populations in skin lesions and peripheral blood (PB) [7–9]. Remarkably, Th17 lymphocytes are increased in the lesional skin and PB, contributing to the disease pathogenesis through IL-17 and IL-22 production [10, 11]. These cytokines promote the recruitment and activation of immune cells and increase keratinocyte proliferation, respectively [12, 13]. Particularly, IL-17 upregulates the expression of TNF*α*, IL-6, and IL-8 by keratinocytes; these cytokines collaborate to maintain the inflammatory process [14, 15].

Importantly, recent evidence obtained from inflammatory conditions demonstrated the presence of two different subsets of Th17 cells, conventional and pathogenic [16]. Conventional Th17 cells are characterized by IL-17 production and expression of the master transcription factor ROR*γ*t. Pathogenic Th17 cells can arise from conventional Th17 cells, changing the cytokine and transcriptional pattern expression [16, 17]. Thus, pathogenic Th17 cells produce IL-17, IFN-*γ*, and TNF*α* simultaneously or only IFN-*γ* and coexpress ROR*γ*t and T-bet [18, 19]. Experimental models in mice such as colitis or experimental autoimmune encephalitis (EAE) have shown an increased frequency of pathogenic Th17 cells. Furthermore, animals transferred with these cells had increased severity and an earlier onset time after the induction of pathology [19, 20].

Pathogenic Th17 cells have been identified in a few human inflammatory pathologies such as Crohn's disease, multiple sclerosis, and inflammatory bowel disease where an increased number of these cells were shown [21–23]. Interestingly, in type 1 diabetes, there is a positive correlation between the percentage of pathogenic Th17 cells and disease severity, suggesting that this population could have an important role in inflammatory autoimmune diseases [24]. In psoriasis, IL-17^+^ IFN-*γ*^+^ CD4^+^ lymphocytes as well as high levels of IL-17, IFN-*γ*, and TNF*α* in the blood could suggest the presence of pathogenic Th17 cells and the existence of a systemic component [10, 25, 26]. In a previous work, we found a high frequency of Th17 cells with a pathogenic phenotype in the lesional skin of patients [27]. However, there is no evidence if this cell phenotype could be related to the severity of psoriasis.

The therapy for psoriasis patients is focused on controlling the inflammatory process through topical or systemic immunosuppressive agents, with the severity of lesions evaluated by the Psoriasis Area and Severity Index (PASI), Body Surface Area (BSA), and Dermatology Life Quality Index (DLQI) that determines the chosen therapy. According to the guidelines for psoriasis treatment, patients with mild severity are treated with topical drugs such as corticosteroids and vitamin D_3_ analogues, whereas severe cases are treated with systemic immunosuppressive agents or with antibodies (biological therapy) against inflammatory cytokines [28–30].

One of the biological therapy strategies is the blockage of TNF*α*. Agents such as etanercept (fusion protein against TNF*α*) showed a reduction of 75% of the PASI score [31], whereas antibodies such as infliximab or adalimumab showed a wide range of PASI percentage reduction, from 50 to 90 [32–35]. Antibodies against IL-17 such as secukinumab, ixekizumab, and bimekizumab showed a reduction up to 90% of the PASI score [36–38]. Despite the efficacy of these treatments, in the clinical practice, it is observed that some patients do not respond or even lose responsiveness to the selected therapy, which could suggest a change in the immune response such as the cytokines involved in the generation of lesions [39].

In this work, we determined the conventional and pathogenic profile of Th17 cells from the skin and peripheral blood of psoriasis patients and evaluated their correlation with disease severity as well as the possible implication of this population for the selection of biological treatment. Our results show that the identification of Th17 cells with pathogenic features in PB is an indicator of disease severity and their assessment could be a potential immunological marker for the selection of biological therapy.

## 2. Materials and Methods

### 2.1. Recruitment of Psoriasis Patients and Healthy Subjects

After approval by the ethics committee of the Hospital de Especialidades Centro Médico Nacional Siglo XXI and Centro Dermatológico Ladislao de la Pascua, we recruited 40 patients with psoriasis diagnosis without topical (at least 15 days) or systemic (two months at minimum) treatment. The study was approved by the ethics committees (protocol R-2007-788-058) of both hospitals and was conducted in accordance with the Helsinki statement. Volunteer patients signed the written informed consent after the protocol aims and procedures were read and explained to them. The treating physician evaluated the severity of the lesions by calculating the PASI, BSA, and DLQI and obtained skin biopsies and peripheral blood. The healthy control skin was obtained from gastrointestinal surgeries nonrelated to autoimmune disorders, cancer, or dermatology affections after written informed consent was signed. Blood from healthy subjects was obtained from buffy coats provided by the Blood Bank of the Hospital de Especialidades Centro Médico Nacional Siglo XXI.

### 2.2. Skin and Blood Sample Collection and Preparation

When possible, both the lesional and nonlesional biopsies (6 mm) from patients were collected of the same anatomical region, considering at least 2 cm between them. In the biopsies from healthy controls, the lesional skin and nonlesional skin were fixed in 4% paraformaldehyde for later histological analysis. Fixed biopsies were dehydrated with a train of solvents including 70% EtOH, 96% EtOH, dehydrated EtOH, and xylene. Tissues were embedded in paraffin, blocks were obtained, and 15 *μ*m sections were placed in charged glass slides (Superfrost Plus Yellow).

Blood samples were collected from each patient through venous puncture, received in tubes with EDTA, and processed for obtaining peripheral blood mononuclear cells (PBMC).

### 2.3. Immunofluorescence Assays and Confocal Microscopy

Remaining paraffin in the tissue was removed by placing the slides into a stove (70°C) for 40 min. Tissues were rehydrated with a train of solvents. Heat-induced antigen retrieval was performed using a citrate buffer pH 6.0 (sodium citrate 10 *μ*M) at 120°C for 20 min. Then, tissue was permeabilized for 2 h with a cocktail including 10 mg/mL bovine serum albumin, 5% horse serum, 0.02% sodium azide, and 0.5% Triton. According to the staining, permeabilized tissue was incubated overnight with primary antibodies anti-CD4 (Sigma Aldrich), anti-IL-17 (RD Systems), and anti-IFN-*γ* (BioLegend). Incubation with secondary antibodies F(ab) anti-rabbit FITC, biotin anti-goat (Jackson ImmunoResearch), and anti-mouse APC (Santa Cruz) was performed for 2 h. When necessary, sections were incubated with Texas Red streptavidin (Jackson ImmunoResearch) for 2 h. When specified, fluorescent conjugated antibodies anti-ROR*γ*t PE (eBioscience) and anti-CD161 PE (BioLegend) were incubated for 2 h. Nuclei were counterstained with Hoechst (Invitrogen) for 10 minutes. Sections were finally mounted with Vectashield (Vector Laboratories) and stored at 4°C until analysis. Micrographs were obtained on a Nikon Ti Eclipse inverted confocal microscope (Nikon Corporation) using NIS Elements v.4.50. Imaging was performed using a 20x (dry, NA 0.8) objective lens. Zoom was performed either at 3.4x or with digital zoom. Images were analyzed using FIJI ImageJ Software.

### 2.4. PBMC Isolation and Activation

A density gradient with Lymphoprep (Axis-Shield) was performed with blood from patients or healthy subjects. Live PBMC obtained were counted with a hemocytometer using the trypan blue assay. 5 × 10^6^ PBMC were stained with CellTrace Violet (CTV, Thermo Fisher Scientific) to track the proliferation of T cells. After that, a maximum of 500,000 cells were placed in a 96-well plate and activated with purified anti-CD3 (1 *μ*g/mL, OKT 3 ATCC) and anti-CD28 (6 *μ*g/mL, GeneTex) for 72 h. When specified PMA/ionomycin (2 *μ*L/mL, stimulation cocktail, eBioscience) was added in the last 6 h of CD3/CD28 stimulation. Cells were cultured in RPMI medium, supplemented with 10% fetal bovine serum (FBS), HEPES, penicillin/streptomycin (Biowest), nonessential amino acids, glutamine (Gibco), ciprofloxacin (Altana), and *β*-mercaptoethanol (Sigma Aldrich) at 37°C in 5% CO_2_. To evaluate intracellular cytokine production, the protein transport inhibitor (2 *μ*L/mL, protein inhibitor cocktail, eBioscience) was added 6 h prior to the end of the activation. After incubation, cells were harvested and processed for intracellular staining.

### 2.5. Intracellular Staining and Flow Cytometry

Harvested PBMC were incubated for 20 minutes with a blocking FACS buffer. Then, Live/Dead Fixable Aqua Dead Cell Stain Kit (Invitrogen) was used to assess cellular viability. The extracellular marker of Th17 cells, CD161 (PE, BioLegend), was stained for 20 minutes. After that, we fixed and permeabilized the cells using the True-Nuclear Factor Buffer Set (BioLegend) for 20 minutes. Intracellular antibody anti-CD4 (APCCy7, BD Bioscience), transcription factor ROR*γ*t (PE, eBioscience), and cytokines IL-17 (Alexa Fluor 488, eBioscience) and IFN-*γ* (PECy7, BioLegend) were incubated for 30 minutes and centrifuged, and cells were suspended in FACS buffer. The cell suspension was acquired in a FACSCanto cytometer (BD Biosciences) and analyzed with FlowJo Software (Tree Star).

### 2.6. Treatment Selection of Psoriasis Patients and Follow-Up

For some patients, we sought to determine cytokine production in order to choose a biological treatment. The treating physician collected the lesional skin and blood samples and determined the PASI score, BSA, and DLQI. With skin samples, an immunofluorescence assay, as previously described, was performed. For IL-17 and IFN-*γ* evaluation, PBMC were obtained, activated, and analyzed as mentioned. After immunological analysis, results were communicated to the dermatologist and the selection of a biological treatment was done. The therapeutic antibodies used were secukinumab (anti-IL-17; induction dose 300 mg, maintenance dose 150 mg s.c), adalimumab (anti-TNF*α*; induction dose 80 mg, maintenance dose 40 mg s.c), and Infliximab (anti-TNF*α*; induction dose 500 mg, maintenance dose 500 mg i.v). Twelve weeks after the induction dose was administrated, the physician evaluated the PASI score, BSA, DLQI, and plaque progression of the patient. A blood sample was also collected and analyzed for cytokine production.

### 2.7. Statistical Analysis

All data are presented as the mean ± SEM (standard error of the mean). Immunofluorescence comparisons were made between cell percentages from healthy controls, nonlesional skin and lesional skin. Flow cytometry comparisons were made between cell percentages from healthy subjects and patients. Statistical analyses were performed applying the Mann-Whitney *U* test for nonparametric data. The correlations between the percentage of cytokines or transcription factor expression and PASI were obtained with the Pearson correlation test. All statistical analyses were performed using Prisma Software (GraphPad). Statistical significance was defined as ^∗^*P* < 0.05, ^∗∗^*P* < 0.01, and ^∗∗∗^*P* < 0.001.

## 3. Results

### 3.1. Th17 Cells in the Lesional Skin Correlate with Psoriasis Severity

**Figure 1:**
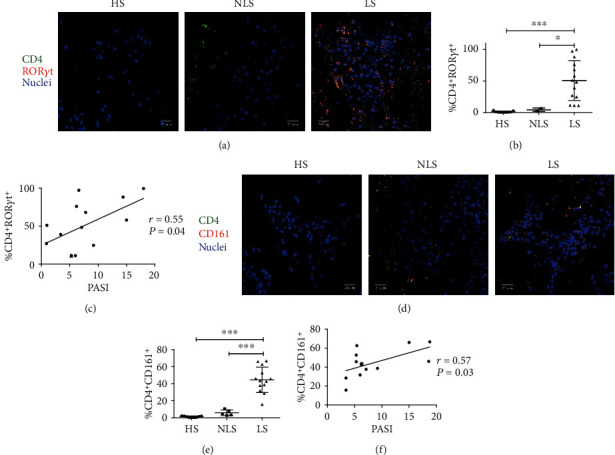
Th17 cells present in the lesional skin correlate with psoriasis severity. Biopsies were obtained from the healthy skin (HS), nonlesional skin (NLS), and lesional skin (LS) and preserved in paraffin. The presence of Th17 cells was evaluated by immunofluorescence using antibodies (a) anti-CD4 (green), anti-ROR*γ*t (red), and (d) anti-CD161 (red). Nuclei were counterstained with Hoechst (blue). Confocal microscopy representative images for (a, d) are shown. (b) Quantification of Th17 cells (CD4^+^ ROR*γ*t^+^) in HS (*n* = 9), NLS (*n* = 2), and LS (*n* = 14), Mann-Whitney *U* test. (c) Spearman's correlation between the Th17 cell (CD4^+^ ROR*γ*t^+^) percentage and the PASI score (*n* = 14). (e) Quantification of Th17 cells (CD4^+^ CD161^+^) in HS (*n* = 9), NLS (*n* = 5), and LS (*n* = 13), Mann-Whitney *U* test. (f) Spearman's correlation between the Th17 cell (CD4^+^ CD161^+^) percentage and the PASI score (*n* = 13). ^∗^*P* < 0.05, ^∗∗∗^*P* < 0.001. Scale bar = 20 *μ*m.

In a previous work, we identified the presence of functional Th17 cells by the coexpression of the IL-17 and the master transcription factor ROR*γ*t in isolated cells from the lesional skin of psoriasis patients [27]. Therefore, the inclusion of the master regulator of Th17 cells would represent a more precise approach to identify and evaluate Th17 cells in psoriasis patients. Here, we evaluated the Th17 cell phenotype in a total of 40 psoriasis patients (Supplementary Table 1). We decided to analyze the presence of Th17 cells (CD4^+^ ROR*γ*t^+^) *in situ* through confocal microscopy. The results show a high presence of Th17 cells (52.7 ± 8%) in the lesional skin (LS) in contrast to the nonlesional skin (NLS) (4.3 ± 2%) or healthy skin (HS) (0.9 ± 0.4%), which is significant (*P* < 0.05, *P* < 0.001; Figures 1(a) and 1(b)). Considering the increment of Th17 cells in LS, we explored if the frequency of these cells could be related to disease severity, determined with the PASI score. Interestingly, we found a positive and significant correlation between PASI and the percentage of Th17 cells in LS (*P* < 0.05, *r* = 0.55; Figure 1(c)).

Previous works show CD161 as a molecule able to identify CD4^+^ IL-17^+^ T cells [40]. Therefore, to confirm our results showed in Figures 1(a) and 1(b), we evaluated the presence of Th17 cells using this strategy. Confocal microscopy images show a high frequency of Th17 cells in LS compared to NLS or HS with a percentage of 44 ± 3.8, 5.8 ± 1.4, and 1.1 ± 0.3, respectively (*P* < 0.001; Figures 1(d) and 1(e)). As observed in Figure 1(c), we found a positive correlation between PASI and the percentage of Th17 cells in LS identified as CD4^+^ CD161^+^ cells, which is significant (*P* < 0.05, *r* = 0.57; Figure 1(f)). These results demonstrate that the lesional skin is highly infiltrated with Th17 cells and their presence correlated with disease severity.

### 3.2. Th17 Cells with the Pathogenic Phenotype Are Increased in the Lesional Skin

It has been reported that in psoriasis patients, *ex vivo* stimulation of dermal cell suspensions shows the presence of Th17 cells with the pathogenic phenotype (CD4^+^ IL-17^+^ IFN-*γ*^+^) [10, 27]. We found that the lesional skin is highly infiltrated with Th17 cells (Figures 1(b)–1(e)); however, the phenotype of these cells *in situ* remains unknown. In order to determine the conventional or pathogenic phenotype, we analyzed the expression of IL-17 and IFN-*γ* in CD4^+^ T cells present in skin biopsies.

**Figure 2:**
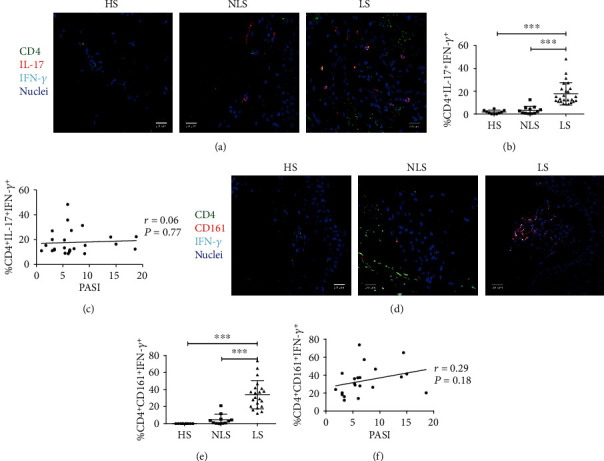
Th17 cells with the pathogenic phenotype are infiltrating the lesional skin of psoriasis patients. Pathogenic Th17 cell identification in healthy (HS), nonlesional (NLS), and lesional (LS) skin biopsies was performed using antibodies anti-CD4 (green) and (a) anti-IL-17 (red) or (d) anti-CD161 (red) along with an anti-IFN-*γ* (cyan). Nuclei counterstaining was performed with Hoechst (blue). Confocal representative images were obtained (a, d). (b) Percentage of pathogenic Th17 lymphocytes (CD4^+^ IL-17^+^ IFN-*γ*^+^) in HS (*n* = 10), NLS (*n* = 12), and LS (*n* = 25), Mann-Whitney *U* test. (c) Spearman's correlation between the percentage of pathogenic Th17 cells (CD4^+^ IL-17^+^ IFN-*γ*^+^) and the PASI score (*n* = 25). (e) Quantification of pathogenic Th17 lymphocytes (CD4^+^ CD161^+^ IFN-*γ*^+^) in HS (*n* = 10), NLS (*n* = 11), and LS (*n* = 21), Mann-Whitney *U* test. (f) Spearman's correlation between the pathogenic Th17 cell (CD4^+^ CD161^+^ IFN-*γ*^+^) percentage and the PASI score (*n* = 21). ^∗∗∗^*P* < 0.001. Scale bar = 20 *μ*m.

Figure 2(a) shows that in NLS and LS, there is a discrete presence of CD4^+^ T lymphocytes producing IL-17 (conventional Th17 cells). Additionally, we observed a significant increase of IL-17^+^ IFN-*γ*^+^ CD4^+^ lymphocytes (pathogenic) in LS (19 ± 2%) compared to NLS (3 ± 1%) and HS (1.7 ± 0.5%) (*P* < 0.001; Figures 2(a) and 2(b)). Despite the high frequency of pathogenic Th17 cells in LS, we did not find a correlation between the percentage of these cells and disease severity (*P* = 0.77, *r* = 0.06; Figure 2(c)). Considering the existence of IL-17^+^/IFN-*γ*^+^ CD4 T cells, we decided to explore the pathogenic features of Th17 cells using a CD161 and IFN-*γ* approach. Micrographs show that LS is highly infiltrated by IFN-*γ*^+^ CD4^+^ CD161^+^ T cells (33 ± 3%) compared to NLS (4.6 ± 1%) or HS where they are practically absent (1.73 ± 0.5%) (*P* < 0.001; Figures 2(d) and 2(e)). Similar to Figure 2(c), the percentage of IFN-*γ*^+^ CD4^+^ CD161^+^ T cells in LS does not correlate with the PASI score (*P* = 0.18, *r* = 0.29; Figure 2(f)). Thus, the lesional skin of patients contains an increased presence of pathogenic Th17 cells albeit not related to disease severity.

### 3.3. Peripheral Blood Th17 Cells Display the Pathogenic Phenotype and Correlate with Disease Severity

**Figure 3:**
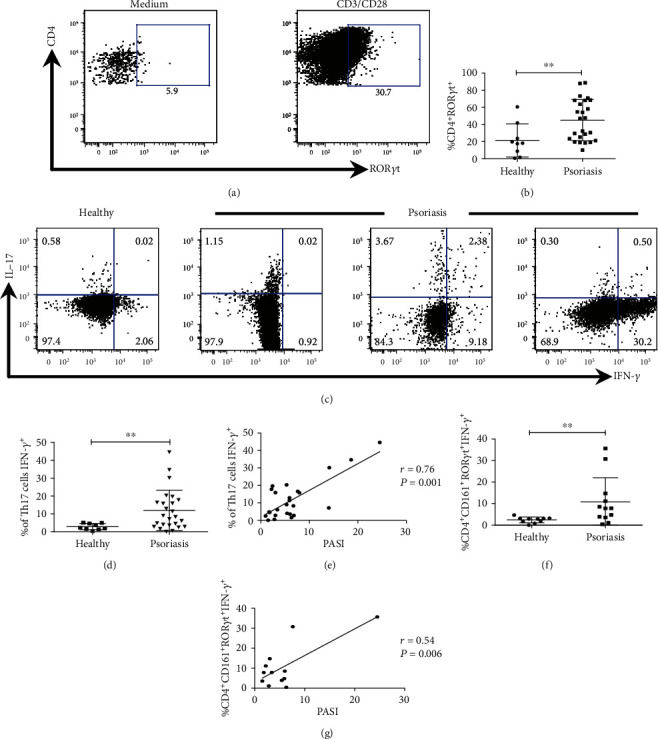
Th17 cells in peripheral blood of psoriasis patients could display conventional or pathogenic phenotype and correlate with the PASI score. PBMC from healthy and psoriasis subjects were stimulated for 72 h with anti-CD3 and anti-CD28 antibodies and analyzed by flow cytometry. (a) Representative plots for Th17 cell (CD4^+^ ROR*γ*t^+^) identification. (b) Quantification of the percentage of Th17 cells (CD4^+^ ROR*γ*t^+^) in healthy (*n* = 9) and psoriasis subjects (*n* = 25), Mann-Whitney *U* test. (c) Representative plots of IL-17 and IFN-*γ* expression by Th17 cells in a healthy subject and three psoriasis patients. (d) Percentage of pathogenic Th17 cells (CD4^+^ ROR*γ*t^+^ IFN-*γ*^+^) in healthy donors (*n* = 9) and psoriasis patients (*n* = 25), Mann-Whitney *U* test. (e) Spearman's correlation between the percentage of pathogenic Th17 cells (CD4^+^ ROR*γ*t^+^ IFN-*γ*^+^) and the PASI score (*n* = 25). (f) Quantification of pathogenic Th17 lymphocytes (CD4^+^ CD161^+^ ROR*γ*t^+^ IFN-*γ*^+^) in healthy (*n* = 10) and psoriasis (*n* = 12) subjects, Mann-Whitney *U* test. (g) Spearman's correlation between the percentage of pathogenic Th17 cells (CD4^+^ CD161^+^ ROR*γ*t^+^ IFN-*γ*^+^) and the PASI score (*n* = 12). ^∗∗^*P* < 0.01, ^∗∗∗^*P* < 0.001.

We demonstrated that Th17 cells are related to disease severity and also there is a high frequency of Th17 cells with the pathogenic phenotype in LS. Psoriasis patients have a systemic presence of IL-17^+^ or IFN-*γ*^+^ T CD4 lymphocytes [25]. However, the production of these cytokines has not been evaluated including the expression of ROR*γ*t. Thus, we determined the potential of Th17 lymphocytes in PB to express IL-17 and IFN-*γ* using ROR*γ*t for their identification (Supplementary Figure 1(a)). Interestingly, in steady state, the CD4^+^ lymphocytes do not express ROR*γ*t; however, after stimulation with anti-CD3/anti-CD28 (CD3/CD28) antibodies, the expression of this transcription factor is observed, which could suggest the presence of a Th17 cell pool (Figure 3(a)). Our next question was to know if the pool of Th17 cells would be different between healthy subjects and psoriasis patients. The results interestingly showed that the percentage of Th17 cells (CD4^+^ ROR*γ*t^+^) is higher in patients than in healthy subjects (Figure 3(b)). These results may suggest that TCR activation promotes the expansion of CD4 lymphocytes imprinted with Th17 features or that T CD4 cells in PB have the potential to give rise to a Th17 cell pool, which seems to be increased in psoriasis patients.

We next evaluated the cytokine production of Th17 cells from PB. After activation of T cells from a healthy donor, we observed the presence of either IL-17^+^ or IFN-*γ*^+^ Th17 cells. Remarkably, when we stimulated PBMC from 25 psoriasis patients, we observed three different cytokine patterns. There were patients whose Th17 cells only produced IL-17 (12%); patients with IL-17, IL-17/IFN-*γ*, and IFN-*γ* expression (12%); and subjects with mainly IFN-*γ* production (76%) (Figure 3(c)).

In healthy donors and psoriasis patients, we found a similar frequency of IL-17^+^ Th17 cells (1.2 ± 0.3% versus 1.6 ± 0.4%) whereas the percentage of IL-17^+^/IFN-*γ*^+^ Th17 cells is increased in patients (0.44 ± 0.1; *P* < 0.05; Supplementary Figure 1(b)). In regard to IFN-*γ*^+^ Th17 cells, we found a higher percentage in psoriasis patients (12.7 ± 2.3) compared to healthy subjects (3 ± 0.62; *P* < 0.01; Figure 3(d)), which indicates the high capacity of PB Th17 cells to acquire pathogenic features in psoriasis patients. Considering these results, the next question was to determine if the acquisition of pathogenic characteristics by Th17 cells could be related to disease severity. Notably, we found a positive and significant correlation between the percentage of IFN-*γ*^+^ Th17 cells and the PASI score (*P* ≤ 0.001, *r* = 0.76; Figure 3(e)), which indicates that at higher PASI scores, the acquisition of pathogenic features by Th17 cells is increased.

To confirm our results, we used CD161 in combination with the Th17 cell identification strategy, showed in Supplementary Figure 1(a), to evaluate the phenotype of Th17 cells and its possible correlation with disease severity. We observed that the percentage of IFN-*γ*^+^ CD161^+^ Th17 cells in psoriasis patients is higher (11.6 ± 3) in comparison to healthy subjects (2.4 ± 0.5; *P* < 0.01Figure 3(f)). Interestingly, Figure 3(g) shows a positive correlation between IFN-*γ*^+^ CD161^+^ Th17 cells and the PASI score (*P* < 0.01, *r* = 0.54). These data confirm the capacity of Th17 cells to acquire pathogenic features. Altogether, our findings exhibit the great potential of the Th17 cell pool to gain pathogenic features in PB, which could be an immunological parameter, associated with the severity of the pathology.

### 3.4. The Th17 Cell Pool Possesses Different Potential to Acquire IL-17 and IFN-*γ* Production in Psoriasis Patients

**Figure 4:**
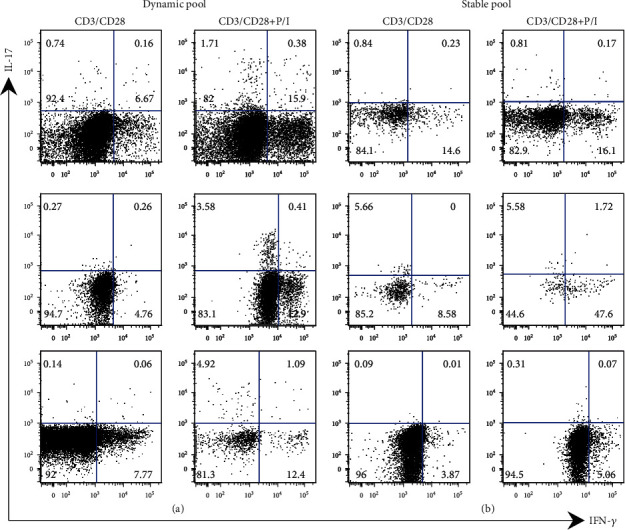
Peripheral Th17 cell pool from psoriasis patients has different cytokine production potential. PBMC from psoriasis patients were stimulated with anti-CD3/CD28 (72 hours) or with anti-CD3/CD28 plus PMA/ionomycin (P/I) (6 hours before the end of the stimulation). IL-17 and IFN-*γ* production by Th17 cells was analyzed through flow cytometry. Representative plots of cytokine pattern expression in the Th17 cell (a) dynamic pool (*n* = 3) or (b) stable pool (*n* = 3). Numbers indicate the percentage of events in each plot quadrant.

The evidence demonstrates that Th17 cells display a pathogenic phenotype under *in vitro* inflammatory conditions [41, 42]. Our data indicate that not all of the Th17 cell pools (CD4^+^ ROR*γ*t^+^ IL-17^−^ IFN-*γ*^−^) in PB from patients acquire the production of IL-17, IFN-*γ*, or both after TCR stimulation (Figure 3(b)). Thus, we asked if the Th17 cell pool was able or not of acquiring the expression of IL-17 and IFN-*γ* after TCR activation plus a strong stimulus such as PMA/ionomycin, which induces several signaling pathways involved in cytokine expression.  Figure 4(a) shows that in a group of patients, TCR activation of the Th17 cell pool induces mainly IFN-*γ* production whereas this stimulation plus PMA/ionomycin increases both IFN-*γ* and IL-17 expression. These results could indicate that in these patients, a strong activation of the Th17 cell pool enables the dynamic capacity to express IL-17.

Nevertheless, we also observed another group of patients with different functional features in the Th17 cell pool.  Figure 4(b) shows that after TCR activation, Th17 cells produce IFN-*γ*, and after stimulation with PMA/ionomycin, they increase the expression of this cytokine with slight IL-17 production. This result could indicate that in some patients, the pool of Th17 cells expressing IFN-*γ* is stable, in spite of a strong activation stimulus. Importantly, the results obtained from PBMC strongly indicate that in psoriasis, the cytokine production by Th17 cells could be different in each patient. In addition, the functional features in the Th17 lymphocyte pool from each patient seem to be a dynamic or stable process.

### 3.5. The Functional Evaluation of Th17 Cells as a Potential Immunological Marker for Biologic Treatment Selection in Psoriasis Patients

The severity of psoriasis is evaluated with three parameters: PASI score, BSA, and DLQI [28]. Importantly, these clinical factors determine if the patient will have a clinical benefit from a biologic treatment. Our results demonstrate that pathogenic Th17 cells are indicators of severity and strongly suggest that in each patient, the Th17 lymphocytes display a diverse cytokine production pattern (Figure 3). Thus, in order to decide if a patient is a candidate to a biologic therapy and which antibody to administrate, we determined the cytokine pattern expressed in Th17 cells before the treatment selection. To this end, we looked for the pathogenic Th17 lymphocytes in the lesional skin biopsies from patients before being treated. In addition, we evaluated PB Th17 lymphocytes after TCR stimulation to know their phenotype. According to the results, an adequate biological treatment for the patient was chosen. After 12 weeks, we analyzed the phenotype of the Th17 cells to know their immunological state.

**Figure 5:**
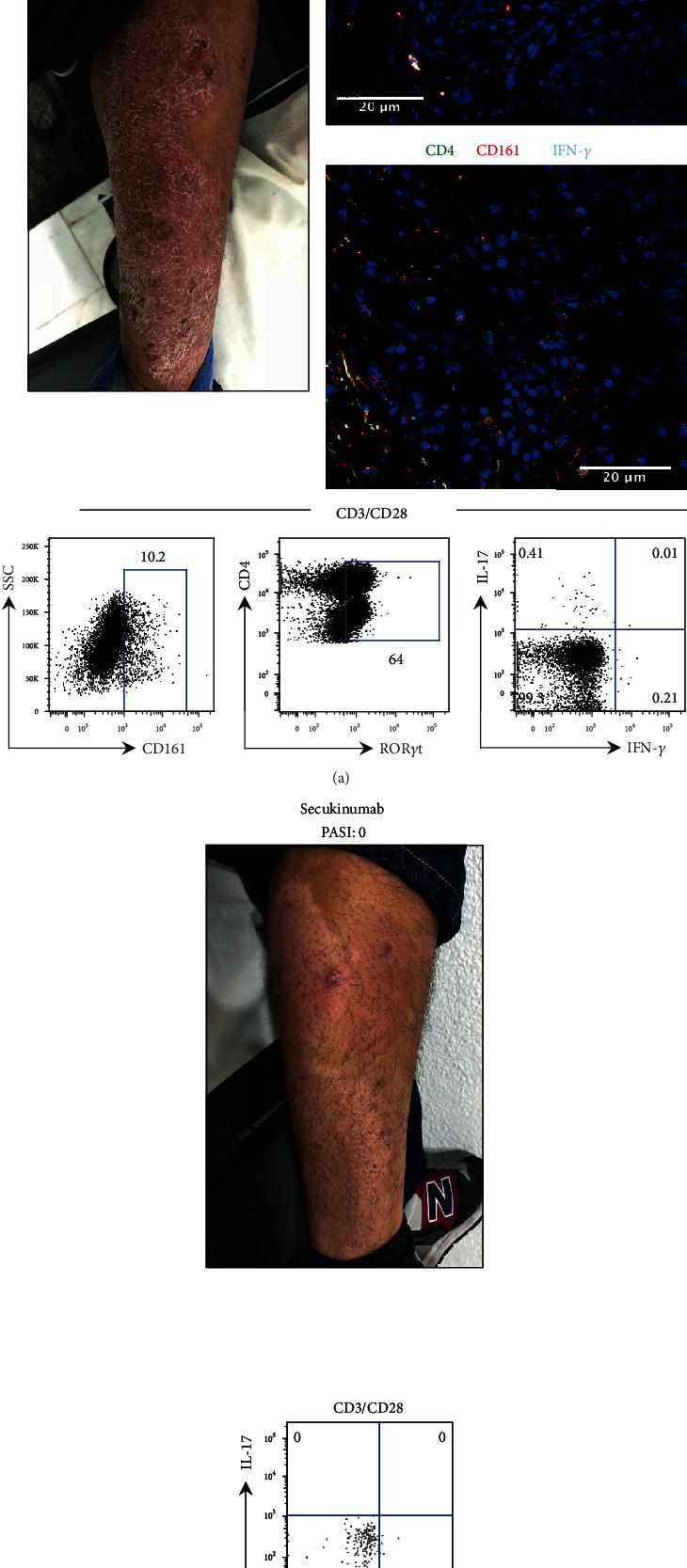
Evaluation of the Th17 cell phenotype is helpful to select the biological treatment of a psoriasis patient. (a) Photography of psoriasis patient P91 (pretreatment, PASI 6.3) shows the most representative skin lesion. LS immunofluorescence staining for Th17 cells using anti-CD4 (green), anti-IFN-*γ* (cyan), anti-IL-17 or anti-CD161 (red), and nuclei (blue). PBMC were stimulated with anti-CD3/CD28. CD161 and ROR*γ*t were evaluated in CD4 T cells by flow cytometry as well as the production of IL-17 and IFN-*γ* in Th17 cells (bottom panels). (b) Photography of a psoriasis patient after secukinumab treatment (PASI 0) taken in the same anatomical site (a). Flow cytometry plot of IL-17 and IFN-*γ* expression in Th17 cells. Scale bar = 20 *μ*m.

Patient P91 had a PASI score of 6.3, BSA 12%, and DLQI of 27, with the presence of plaques in arms and legs. Confocal microscopy images from LS show the presence of CD4^+^ IL-17^+^ IFN-*γ*^+^ and CD4^+^ CD161^+^ cells (Th17 cells), and we did not observe IFN-*γ*^+^ Th17 cells (Figure 5(a)). The results obtained from PB under TCR stimulation show a high percentage of CD161 and ROR*γ*t in CD4^+^ T cells (Th17 lymphocytes), and these cells produced only IL-17. Taking together these results, we decided to administrate secukinumab, which targets interleukin 17. After 12 weeks of therapy, the patient showed great improvement, reaching a PASI score of 0, BSA of 0%, and DLQI of 1; the psoriatic skin lesions disappeared; and Th17 cells did not express IL-17 (Figure 5(b)). This indicates that the immunological analysis of Th17 cells from PB could be relevant to the selection of a therapeutic antibody anti-IL-17.

**Figure 6:**
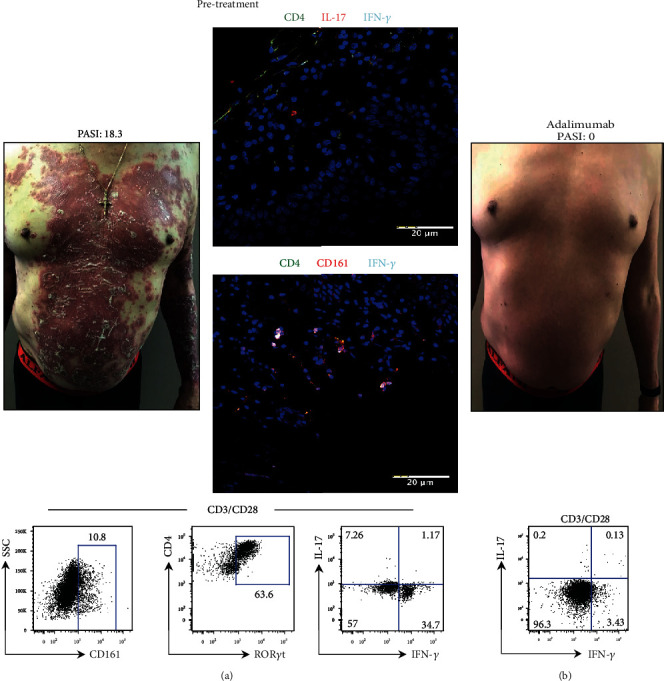
Determination of the pathogenic phenotype of Th17 cells in a psoriasis patient is useful to choose the biological treatment. (a) Prior to treatment, an integral analysis of Th17 cells was performed in patient P52 (PASI 18.3). The photograph shows the most significant psoriatic lesion. Microscopy images show the evaluation of Th17 cells in the lesional skin using antibodies anti-CD4 (green), anti-IFN-*γ* (cyan), anti-IL-17 or anti-CD161 (red), and nuclei (blue). Flow cytometry analyses of CD161 and ROR*γ*t expression in CD4 cells and production of IL-17 and IFN-*γ* in Th17 cells after stimulation. (b) Psoriasis patient photograph after adalimumab administration (PASI 0). Evaluation of IL-17 and IFN-*γ* expression in Th17 cells by flow cytometry (bottom plot). Scale bar = 20 *μ*m.

Patient P52 presented plaques in the trunk and arms, PASI score of 18.3, BSA of 61%, and DLQI of 27. The analyses of microscopy images from LS revealed the presence of pathogenic Th17 cells (CD4^+^ CD161^+^ IFN-*γ*^+^), and additionally, we found some Th17 cells (CD4^+^ IL-17^+^ and CD4^+^ CD161^+^) (Figure 6(a)). The CD4^+^ CD161^+^ T cells from PB displayed an increased frequency of ROR*γ*t, and these Th17 cells showed a pathogenic phenotype due to high interferon gamma expression in response to TCR stimulation (Figure 6(a)). Evidences in human inflammatory conditions show that anti-TNF*α* administration induces a significant amelioration of the disease and diminished IFN-*γ* production [41, 43]. Taking into account our results and published evidence, we chose adalimumab (monoclonal antibody against TNF*α*) as the treatment for this patient. Interestingly, after 12 weeks of treatment, the patient reached a PASI score of 0, BSA of 0%, and DLQI of 2; his lesions became imperceptible; and moreover the expression of IFN-*γ* in Th17 cells was considerably diminished (Figure 6(b)). This data shows that determination of IFN-*γ* production in the skin and in Th17 cells from PB could be useful to select an adequate antibody therapy.

**Figure 7:**
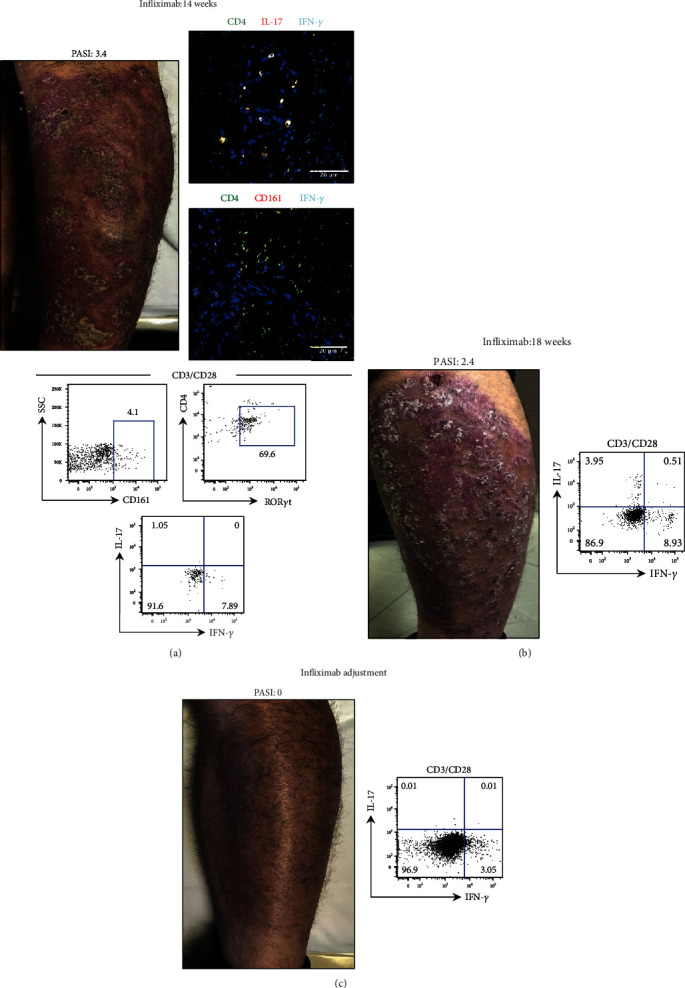
Determination of the pathogenic phenotype of Th17 cells in a psoriasis patient. Consecutive immunologic surveillance is relevant to choose the biological therapy. Integral analysis of Th17 cells IL-17 and IFN-*γ* expression in the lesional skin shown in immunofluorescence panels (CD4: green; IFN-*γ*: cyan; IL-17 or CD161: red; and nuclei: blue) and in peripheral blood (cytometry plots) from patient P21. (a) After infliximab treatment for 14 weeks, the patient presented a PASI score of 3.4 and displayed psoriatic skin lesions (photography). (b) After infliximab treatment (18 weeks), the PASI score was 2.4. Photography shows the lesional skin. (c) A psoriasis patient following infliximab dose adjustment (PASI 0). Picture illustrates the same anatomical site shown in (a–c). Scale bar = 20 *μ*m.

Patient P21 was diagnosed with psoriasis and presented a PASI score of 15.4, BSA of 13%, and DLQI of 20. The patient was treated with infliximab (TNF*α* neutralizing antibody) showing a good response. However, leg lesions persisted after 14 weeks of treatment, maintaining a PASI score of 3.4. As the efficacy seemed to be diminished, immunological analysis was performed in order to know the cytokine production by Th17 cells. The microscopy analyses showed that LS contained CD4^+^ CD161^+^ Th17 lymphocytes and IL-17^+^ IFN-*γ*^+^ CD4^+^ cells. In a similar fashion to Figure 5(a) and Figure 6(a), the percentage of CD4^+^ CD161^+^ ROR*γ*t^+^ lymphocytes increased, and these Th17 cells also expressed IFN-*γ* when stimulated with anti-CD3/CD28 antibodies (Figure 7(a)). In light of these results, the patient continued with infliximab treatment for four more weeks. At this point, the PASI score was 2.4 and the leg lesions were still present (Figure 7(b)). According to the guidelines, in this particular case, modification of the treatment is strongly recommended either by adjusting the dose or by changing the antibody drug [29]. In order to complement the clinical findings, we considered that the assessment of the cytokine profile in Th17 cells would provide an immunological evidence to choose whether to continue with infliximab or to change to another biologic agent. Remarkably, we observed that Th17 lymphocytes from PB acquired IL-17 expression; however, IFN-*γ* production was maintained. Thus, we opted to shorten the interval of infliximab administration.

Figure 7(c) shows that after treatment adjustment, the therapy seemed to be successful considering the reduction of the PASI score to 0, BSA of 0%, and DLQI of 2, as well as the absence of psoriatic lesions. In regard to the Th17 cell phenotype, we observed a diminished production of IFN-*γ* and no more IL-17 expression.

These results suggest that not only the cytokine evaluation is important for the treatment selection but also the immunological follow-up could be relevant to determine the adequate modification for the therapy regimen.

## 4. Discussion

Psoriasis is an inflammatory autoimmune skin disease where Th17 lymphocytes, through IL-17, contribute to the disease pathogenesis. The presence of pathogenic Th17 lymphocytes has been related to the severity on mouse autoimmune models and patients with type 1 diabetes [24]. Here, we demonstrated that the presence of Th17 lymphocytes with pathogenic features in PB is related to disease severity and can be a potential marker to decide if a patient would be a candidate to receive biological treatment as well as a contributor on choosing the adequate antibody.

Our results showed similar expression patterns and high percentages of Th17 cells identified either as CD4^+^ ROR*γ*t^+^ or CD4^+^ CD161^+^ cells in the skin lesions of psoriasis patients. CD161 has been reported as an identification marker of Th17 cells in psoriasis patients [40]. However, this is the first approach of identification using ROR*γ*t. According with the key role of ROR*γ*t in the biology of Th17 cells [44], we consider that its evaluation provides a reliable strategy for the identification of this population. Importantly, our results indicate that among the cells present in the skin, Th17 cells compose a large part of the T cell populations infiltrating psoriasis lesions.

Although it is extensively accepted that Th17 cells are increased in psoriasis patients [45, 46], a correlation with severity was not previously reported. Interestingly, we found a positive and significant correlation between the percentage of Th17 lymphocytes in PB (CD4^+^ ROR*γ*t^+^ or CD4^+^ CD161^+^) and the PASI score. These findings strongly support the idea that this population is implicated in the pathogenesis of psoriasis. It would be probable that previous strategies for Th17 cell identification based on IL-17 expression might have underestimated the real frequency of these cells considering that not all of Th17 cells are producing this cytokine.

Additionally, we observed that LS is highly infiltrated by cells with the pathogenic phenotype (IL-17^+^ IFN-*γ*^+^ or CD161^+^ IFN-*γ*^+^). This cytokine pattern might be expressed by a portion of the Th17 cells present in LS due to the inflammatory microenvironment [47], which can induce the polarization towards a pathogenic phenotype. In addition to the high production of cytokines relevant to psoriasis, such as of IFN-*γ* and TNF*α* [16], pathogenic Th17 cells display several features that could contribute to the physiopathology of autoimmune diseases. For instance, it has been reported that pathogenic Th17 lymphocytes have a higher rate of proliferation, diminished apoptosis capacities, and resistance to regulation by IL-10 [48]. Importantly, they are refractory to glucocorticoids, which are proposed to be the mainstay topical anti-inflammatory drugs [49, 50]; this brings the possibility that the presence of pathogenic Th17 cells could be implicated in the resistance to this therapy.

Interestingly, we did not find a correlation between PASI and the percentage of pathogenic Th17 cells in the skin. This could be explained by the lower frequency of cells producing cytokines compared to the higher presence of Th17 cells or by the variability of the Th17 phenotypes. However, the high presence of IL-17 and IFN-*γ* expression by pathogenic Th17 cells in the lesional skin strongly suggests the contribution of these cytokines to the intricate inflammatory process.

Inflammatory cytokines have been detected in peripheral blood of psoriasis patients, which indicates the existence of a systemic compartment [51]. Interestingly, we observed an increment in the percentage of Th17 cells (CD4^+^ ROR*γ*t^+^) in PB after TCR activation and their frequency is higher in patients compared to healthy subjects. This suggests that patients could have a larger pool of Th17 cells, which might be polarized towards a pathogenic phenotype in response to the inflammatory systemic microenvironment. Evidence from mouse models has shown that pathogenic Th17 cells can diminish or lose ROR*γ*t expression. In contrast, in all the patients that we analyzed, the expression of this transcription factor was remarkably higher. It is reported that the maximum expression of ROR*γ*t mRNA is at 16 hours [44]. Considering that we performed this set of experiments at 72 hours of activation, it is probable that pathogenic Th17 cells from PB keep expressing ROR*γ*t.

Our results also show heterogeneity in the cytokine expression in Th17 cells from psoriasis patients. Interestingly, we could classify the patients in three groups based on the cytokine pattern produced by Th17 cells (IL-17^+^, IL-17^+^/IFN-*γ*^+^, and IFN-*γ*^+^). It has been proposed that in some psoriasis patients, the disruption of the skin barrier integrity could promote translocation of the microbiota to PB [52], conferring systemic inflammatory alterations that could contribute to the heterogeneity observed in Th17 cells. In psoriasis patients, a high presence of inflammatory dendritic cells that through IL-1*β* and IL-23 production can polarize Th17 cells towards a pathogenic phenotype has also been described (IL-17 and IFN-*γ* production) [53]. This suggests that the inflammatory processes or Th17 cell priming could be different, and this might be translated into the differential cytokine patterns observed in Th17 cells. Our results also show the existence of a Th17 cell pool with dynamic production of IL-17 and IFN-*γ* after a strong stimulation and another pool with stable IFN-*γ* production. This result could provide another explanation of why we observed three different cytokine production patterns in patients.

Evidence shows that T cells from patients produce more IL-17 than healthy subjects in a PMA/ionomycin activation context [25, 46]. In contrast, under an anti-CD3/CD28 stimulation, we observed that IL-17 produced by Th17 cells was similar between healthy subjects and patients, which could be attributed to the different nature of the stimuli employed. Remarkably, our data shows that activation via TCR promotes increased acquisition of a pathogenic phenotype in Th17 cells from psoriasis patients, revealing that their Th17 cell pool could be prone to gain this phenotype. Evidence in mice has shown that Th17 lymphocytes possess epigenetic marks, particularly in the *ifng* locus, which promote its expression under inflammatory conditions [54, 55]. Thus, it is possible that Th17 cells from psoriasis patients have a different epigenetic landscape compared to healthy donors.

One of the most important findings in this work is the positive correlation between PB IFN-*γ*^+^ Th17 cells and the PASI score, which indicates that these cells are associated with the severity. In psoriasis, the existence of biomarkers associated with severity, such as IL-36*γ*, Squamous Cell Carcinoma Antigen 2 (SCCA2), and miRNA1266, has been reported [56–58]. To our knowledge, this is the first report of a Th17 cells subset associated to the severity of psoriasis.

The treatment of psoriasis patients is performed following clinical guidelines. This set of documents recommends the usage of topic, systemic, and biological therapies based on PASI, BSA, and DLQI [28–30]. Currently, there are no immunological parameters that could provide additional evidence to decide if a patient is a candidate to receive biologic therapy, which antibody agent to use, and whether the treatment should continue or change. Interestingly, we present three representative cases where we performed an integral analysis focused on Th17 cells. This immunological assessment could provide critical information to support clinical decisions regarding the biologic therapy.

The immunological assessment of patient P91 identified Th17 cells producing IL-17. Taking together this result and the clinical parameters led us to decide that this patient would be a candidate to receive an antibody therapy, with anti-IL-17 antibody a rational option. Amelioration of the disease in this patient demonstrated that the pathology was in fact driven by IL-17.

On the other hand, we found a high percentage of Th17 cells producing IFN-*γ* in PB and LS from patient P52. In addition, this patient had a high PASI score (18.3), which is consistent to the correlation observed in Figure 3. In this case, biologic therapy is strongly recommended based on the high severity. Due to the IFN-*γ* expression, an anti-IFN-*γ* antibody would be the reasonable option for the treatment. Nevertheless, it has been reported that an anti-IFN-*γ* therapy might be not effective for psoriasis patients [59]. Works in mice and humans have shown that TNF*α* could promote the IFN-*γ* expression in Th17 cells [60]. Therefore, we decided that administration of adalimumab could be a better treatment option. These results strongly suggest that evaluation of IL-17 and IFN-*γ* in Th17 cells could be considered an essential immunological marker, to decide if a patient could be a candidate to receive biologic therapy and aid in choosing an adequate blocking antibody. However, we considered that this immunological evaluation must be performed on a higher number of patients in order to validate our results.

Our findings also highlight the necessity to know the cytokine pattern that a specific patient is producing. If a therapeutic antibody is administrated in a patient who does not produce its specific cytokine target, the therapy could be inefficient. We consider that these findings are relevant because of the implications that a wrong therapy could cause. For example, it is known that biologic agents might promote the generation of antidrug antibodies that will affect the efficacy of a subsequent biologic therapy.

The guidelines recommend the course of action if a patient is not responsive to the biologic therapy [61]. Our results also suggest that an immunological assessment would be useful to complement the clinical findings in order to decide whether a treatment should be continued or changed. Compared to the proposed biomarkers in psoriasis, the evaluation of pathogenic features in Th17 cells could be applied to the follow-up of patients that do not have or lose responsiveness to the treatment, as observed in patient P21.

Our findings point to the value of evaluating immunological parameters such as the cytokines expressed by the Th17 cells in psoriasis patients. This data in addition to the clinical guideline recommendations could be of great importance for the selection of an adequate biological therapy.

## 5. Conclusions

In this work, we reported that the Th17 cell pathogenic phenotype is increased in the lesional skin and peripheral blood of psoriasis patients. Remarkably, we demonstrated that Th17 cells in the skin and particularly the pathogenic phenotype in peripheral blood correlate with the disease severity. Our results also exhibit that the evaluation of the immunological state of the Th17 phenotype could be a potential immunological marker to indicate if a patient can be a candidate for biological therapy, which is the adequate biologic drug, or if the antibody should be changed or not. Finally, our findings highlight the importance of including an immune personalized analysis approach to support or provide additional information for therapy selection in the clinical practice.

## Data Availability

The data used to support the findings of this study are available from the corresponding author upon request.

## Supplementary Materials

Supplementary Materials Table 1: demographic data of psoriasis patients. Figure 1: cytometry identification strategy and quantification of the Th17 cell phenotype.
