# Supplementary material for: Association of Pathogenic Th17 Cells with the Disease Severity and Its Potential Implication for Biological Treatment Selection in Psoriasis Patients
Source: Mediators Inflamm. 2020 Jul 31;2020:8065147. doi: 10.1155/2020/8065147 (PMC7415124; doi:10.1155/2020/8065147)
Supplement: Supplementary Materials — Table 1: demographic data of psoriasis patients. Figure 1: cytometry identification strategy and quantification of the Th17 cell phenotype. [file 8065147.f1.pdf]

Supplementary Materials

Table 1. Demographic data of psoriasis patients.

Patient	Sex	Age	PASI
**1**	Male	61	7.9
**2**	Female	28	3
**3**	Female	34	6.7
**4**	Male	39	14.1
**5**	Female	61	5.3
**6**	Female	38	1
**7**	Male	62	5.3
**8**	Male	37	5.9
**9**	Male	55	14
**10**	Female	41	6.8
**11**	Male	55	3.4
**12**	Male	50	6
**13**	Male	66	5.4
**14**	Male	58	5.9
**15**	Female	56	2.8
**16**	Female	70	24.5
**17**	Male	69	7.6
**18**	Male	36	6.3
**19**	Male	21	3
**20**	Female	23	1.8
**21**	Female	52	2.2
**22**	Female	22	1.5
**23**	Female	38	1.7
**24**	Male	63	18.3
**25**	Male	54	10.6
**26**	Female	42	2.4
**27**	Male	46	6.2
**28**	Female	36	18
**29**	Female	46	15
**30**	Male	45	7.8
**31**	Female	80	6.6
**32**	Male	63	5.3
**33**	Male	26	7.1
**34**	Female	57	9.2
**35**	Male	51	6
**36**	Male	56	18.8
**37**	Male	55	14
**38**	Male	31	3

**39**	Male	37	3
**40**	Female	47.0	2.3

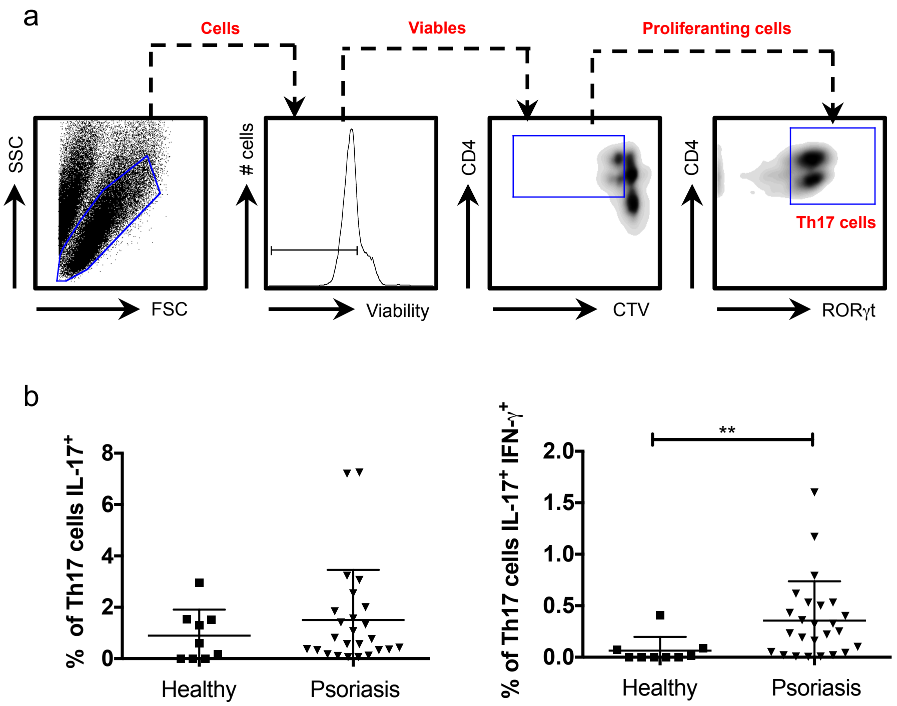

Supplementary Figure 1. Cytometry identification strategy and quantification of Th17 cells phenotype. (a) Representative plots of the gating strategy of Th17 identification. SSC vs FSC plot shows the mononuclear cells area selection, then cell viability and proliferation are assessed. Finally, coexpression of CD4 and the transcription factor RORγt identifies the Th17 subset. Quantification of (b) conventional Th17 lymphocytes ($CD4^{+}$ $ROR\gamma t^{+}$ $IL\text{-}17^{+}$) and (c) pathogenic Th17 cells ($CD4^{+}$ $ROR\gamma t^{+}$ $IL\text{-}17^{+}$ $IFN\text{-}\gamma^{+}$) in healthy donors (n=9) and psoriasis patients (n= 25) Mann Whitney test. **p<0.01
